# Impaired Periodontitis-Induced Cytokine Production by Peripheral Blood Monocytes and Myeloid Dendritic Cells in Patients with Rheumatoid Arthritis: A Case–Control Study

**DOI:** 10.3390/jcm13175297

**Published:** 2024-09-06

**Authors:** Daniela S. Silva, Paula Laranjeira, Ana Silva, Isabel Silva, Marta Kaminska, Piotr Mydel, Charlotte de Vries, Karin Lundberg, José António P. da Silva, Isabel P. Baptista, Artur Paiva

**Affiliations:** 1 Periodontology Institute, Faculty of Medicine, University of Coimbra, 3000-548 Coimbra, Portugal; 2 Group of Environmental Genetics of Coimbra Oncobiology (CIMAGO), Institute for Clinical and Biomedical Research (iCBR), Faculty of Medicine, University of Coimbra, 3000-548 Coimbra, Portugal; 1979paula@gmail.com (P.L.); jdasilva@ci.uc.pt (J.A.P.d.S.); artur.paiva@chuc.min-saude.pt (A.P.); 3 Flow Cytometry Unit, Department of Clinical Pathology, Hospitais da Universidade de Coimbra, Unidade Local de Saúde de Coimbra (ULS), 3004-561 Coimbra, Portugal; 4 Center for Innovative Biomedicine and Biotchnology (CIBB), University of Coimbra, 3000-548 Coimbra, Portugal; 5 Clinical Academic Center of Coimbra (CACC), 3004-504 Coimbra, Portugal; 6 Center for Neuroscience and Cell Biology (CNC), University of Coimbra, 3004-504 Coimbra, Portugal; 7 Broegelmann Research Laboratory, Department of Clinical Science, Faculty of Medicine and Dentistry, University of Bergen, 5021 Bergen, Norway; marta_kaminska@outlook.com (M.K.); piotr.mydel@uib.no (P.M.); 8 Department of Microbiology, Faculty of Biochemistry Biophysics and Biotechnology, Jagiellonian University, 30-387 Krakow, Poland; 9 Department of Medicine Solna, Division of Rheumatology, Karolinska Institute, 17164 Solna, Sweden; charlotte.de.vries@ki.se (C.d.V.); karin.lundberg@ki.se (K.L.); 10 Rheumatology Department, Hospitais da Universidade de Coimbra, Unidade Local de Saúde de Coimbra (ULS), 3004-561 Coimbra, Portugal; 11 Ciências Biomédicas Laboratoriais, Instituto Politécnico de Coimbra, ESTESC-Coimbra Health School, 3046-854 Coimbra, Portugal

**Keywords:** periodontitis, rheumatoid arthritis, monocytes, dendritic cells, flow cytometry

## Abstract

**Background**: Immune cells from rheumatoid arthritis (RA) patients display a reduced in vitro response to *Porphyromonas gingivalis* (*P. gingivalis*), which may have functional immune consequences. The aim of this study was to characterize, by flow cytometry, the frequency/activity of monocytes and naturally occurring myeloid dendritic cells (mDCs) in peripheral blood samples from patients with periodontitis and patients with periodontitis and RA. **Methods**: The relative frequency of monocytes and mDCs in the whole blood, the frequency of these cells producing TNFα or IL-6 and the protein expression levels for each cytokine, before and after stimulation with lipopolysaccharide (LPS) from *Escherichia coli* plus interferon-γ (IFN-γ), were assessed by flow cytometry, in peripheral blood samples from 10 healthy individuals (HEALTHY), 10 patients with periodontitis (PERIO) and 17 patients with periodontitis and RA (PERIO+RA). **Results**: The frequency of monocytes and mDCs producing IL-6 or TNF-α and the expression of IL-6 and TNF-α in the PERIO group were generally higher. Within the PERIO+RA group, *P. gingivalis* and related antibodies were negatively correlated with the monocyte and mDC expression of IL-6. A subgroup of the PERIO+RA patients that displayed statistically significantly lower frequencies of monocytes producing IL-6 after activation presented statistically significantly higher peptidylarginine deiminase (PAD)2/4 activity, anti-arg-gingipain (RgpB) IgG levels, mean probing depth (PD), periodontal inflamed surface area (PISA) and bleeding on probing (BoP). **Conclusions**: In the patients with PERIO+RA, innate immune cells seemed to produce lower amounts of pro-inflammatory cytokines, which are correlated with worse periodontitis-related clinical and microbiological parameters.

## 1. Introduction

Inflammation is a physiological response to infection or injury aimed at promoting tissue and overall homeostasis. Inflammation not only forces a transient impair in tissue function, but, in turn, can contribute to the pathogenesis of several systemic non-communicable diseases, such as rheumatoid arthritis (RA) and periodontitis [1]. Knowledge of how immune mechanisms and inflammatory responses are regulated is critical for understanding the pathogenesis and the interplay of such complex diseases.

Periodontitis, a destructive inflammatory disease affecting the supporting tissues of the teeth, is the most prevalent bacteria-driven chronic inflammatory disease in humans [2]. The disease’s onset and progression can span decades, and when left untreated, it leads not only to tooth loss, but also to masticatory impairment and negative influences on the patient’s quality of life [3,4,5]. The tissue destruction seen in periodontitis is the result of interactions between pathogenic bacteria, namely *Porphyromonas gingivalis* (*P. gingivalis*), and the host immune response. *P. gingivalis*, a keystone periodontal pathogen, is a highly adapted bacteria and is equipped with numerous virulence factors, including fimbriae, lectin-type adhesins, a polysaccharide capsule, lipopolysaccharides (LPS), potent proteinases, metabolic toxins, hemagglutinins, and various enzymes that facilitate the evasion of host immune defenses and the destruction of periodontal tissue.

RA is a chronic, systemic and autoimmune disease, characterized by an inflammatory process in the synovial joints [6]. The current pathogenetic paradigm of RA suggests that the disease is triggered by a complex interaction of genetic, environmental and hormonal factors that break immune tolerance and lead to the production of anti-citrullinated protein antibodies (ACPAs) [7]. Mucosal challenges have been suggested to play an important role in this matter, with an emphasis on smoking and periodontitis [8,9]. Various inflammatory conditions encompass increased citrullination, a post-translational protein modification by the peptidylarginine deiminase enzyme (PAD). However, the production of ACPAs is essentially restricted to RA patients [10]. Out of five mammalian PAD isozymes, PAD2 and PAD4 are the most relevant in RA pathogenesis because they are predominantly overexpressed in immune cells, including macrophages and neutrophils [11]. Despite extensive evidence linking citrullination and RA, the mechanisms responsible for regulating PAD activity remain poorly understood.

It has been suggested that the link between RA and periodontitis depends on *P. gingivalis* [12,13], which is the only bacterial strain that expresses its own PAD enzyme [12,13]. Moreover, there is substantial evidence to suggest that periodontitis triggers a systemic inflammatory response, and this could explain its association with an increased incidence of systemic health outcomes [14,15,16], including the onset and progression of RA [17].

Monocytes acquire macrophage-like features while in circulation in the blood, so these cells can be channels by which systemic inflammation is increased during periodontal disease. Dendritic cells (DCs), on the other hand, as ‘professional’ antigen-presenting cells, must maintain fine control of lysosomal degradation for the limited processing of internalized antigens (to preserve antigenic epitopes) [18]. This property of DCs is a particular strength for initiating immunity to viruses and tumors, but it is a vulnerability when faced with invasive pathogens such as *P. gingivalis*. A study [19] that stimulated monocyte-derived DCs from RA patients with *P. gingivalis* suggested an immunologic link between *P. gingivalis* and RA. According to this study, immune cells from RA patients display a reduced response to *P. gingivalis*, which has functional consequences for the immune response. Moreover, since dendritic cells are highly migratory, they may promote microbial dissemination to other distant sites [20].

As far as we know, no studies exist addressing this issue in patients with concomitant periodontitis and RA, with full clinical and microbiological characterization and when analyzing naturally occurring dendritic cells. The aim of the present study was to characterize, by multi-color flow cytometry, the frequency and activity of circulating monocytes and dendritic cells in peripheral blood samples from patients with periodontitis plus RA, periodontitis patients and healthy individuals, in order to understand the immune status of these patients. Furthermore, the study is intended to obtain evidence about hypothetical correlations between the changes in the proportion or functions of these immune cell populations and clinical and microbiological data, for application in clinical diagnosis and the future development of treatments.

## 2. Materials and Methods

### 2.1. Study Design and Setting

This was a registered (ISRCTN 17950307) case–control study conducted, according to the guidelines of the Declaration of Helsinki, at Centro Hospitalar e Universitário de Coimbra, Coimbra (CHUC), Portugal, from November 2018 to October 2021. All procedures were approved by the hospital’s ethics committee (protocol number CHUC 130-17). All participants provided written informed consent prior to inclusion in the study.

### 2.2. Patient Recruitment and Selection

Three groups of participants were included: (1) patients with periodontitis and RA (PERIO+RA) recruited among consecutive adult individuals with RA (ACR/EULAR 2010 criteria [21] attending CHUC Rheumatology Department; (2) patients with periodontitis (PERIO) recruited among individuals attending the Periodontology appointment of Faculty of Medicine from University of Coimbra, Coimbra, Portugal; and (3) healthy subjects (HEALTHY), recruited from appointments at Dentistry Department of FMUC for issues other than periodontology.

All consenting RA patients underwent an oral screening examination by an experienced periodontologist (DSS) and the Periodontal Screening and Recording (PSR) index was established [22]. Subjects presenting PSR scores of 3 in two or more sextants, or a PSR score of 4 in any sextant, were submitted to a full periodontal assessment. The full periodontal assessment allowed the classification of periodontitis according to its severity (Stages I–IV), extent and disease progression (grade A, B, or C). Only patients with moderate and severe periodontitis (Stages ≥ II) were included in the PERIO+RA group, irrespective of the extent and grade. Recruitment continued until seventeen patients were included.

Ten systemically healthy patients with periodontitis (PERIO), matched for age and gender to the PERIO+RA group, were recruited among stage II, III and IV periodontitis patients.

Ten systemically healthy participants without periodontitis were recruited as control group (HEALTHY).

Additional exclusion criteria were as follows: fewer than six natural teeth, current inflammatory conditions other than RA, history of infection/antibiotic use in the previous four months, history of periodontal treatment, changes in RA medication in the previous three months, treatment with biologic disease-modifying antirheumatic drugs (bDMARDs), current or prior smoking habits, diabetes, current pregnancy, or lactation.

### 2.3. Periodontal Assessment

Full periodontal assessment was performed by DSS to quantify the current periodontal inflammation. This was established according to the following parameters, all measured at six sites per tooth:

- Probing depth (PD): The distance, in millimeters, from the gingival margin to the deepest point of the periodontal pocket/sulcus. Each patient is represented by the mean PD measured at all sites.
- Gingival recession: The distance, in millimeters, from the cement–enamel junction to the gingival margin. PD and gingival recession were measured with a straight periodontal probe (PCP15, Hu-Friedy, Chicago, IL, USA).
- Bleeding on probing (BoP): the percentage of sites that exhibit bleeding at 30 s after probing.
- Plaque index (PI): the percentage of tooth sites exhibiting dental plaque.

Combined indices:

- Clinical attachment loss (CAL): Scores for PD and gingival recession were summed to retrieve the CAL at each site. Each patient was represented by the mean CAL of all site measures.
- Maximum PD and Maximum CAL: the arithmetic mean of the two highest PD and CAL site measurements.
- Periodontal inflamed surface area (PISA): an estimation of the total periodontal inflamed area, in square millimeters, based on the six-point PD measurements and presence or absence of BoP, processed by a freely downloadable spreadsheet [23].

Periodontal diagnosis and classification were established after agreement between the examiner and a second experienced periodontologist (IPB). The severity of disease (stages I–IV) was defined using a consensual matrix (severity: inter-dental CAL and missing teeth; complexity: PD and furcation involvement) [24]. The extent of the disease was defined as localized (affecting <30% of the teeth) or generalized (≥30% of the teeth).

The progression of periodontitis was also assigned using the assessment of bone loss/age index [24] (grade A: <0.25; B: 0.25–1.0; C: >1.0). Since diabetes and smoking habits were defined as exclusion criteria, no grade modifiers were considered.

### 2.4. Rheumatological Assessment

Two experienced rheumatologists, blinded to periodontitis status (JR, SS), examined each RA patient. Disease activity score for 28 joints (DAS28) was calculated using three variables (number of tender and swollen joints and CRP levels (mg/L) [25]) to quantify RA disease activity.

### 2.5. Sampling

All subjects underwent a peripheral blood sample collection by venipuncture to serum tubes and lithium heparin tubes.

Serum aliquots, obtained after blood clotting and centrifugation, were stored at −80 °C, until analyzed for CRP levels, anti-cyclic citrullinated peptide (CCP2) IgG, PAD2 and PAD4 activity (PAD2_Act_ and PAD4_Act_) and antibody levels for *P. gingivalis*-virulence-associated cysteine proteinase RgpB (Arg-gingipain) and Kgp (Lys-gingipain).

Subgingival biofilm samples of all periodontitis patients (PERIO and PERIO+RA groups) were taken. Samples were collected from the two highest probing depth (PD) sites using sterile curettes and placed into two different test tubes containing 200 µL of RNA. Later, they were snap-frozen in liquid nitrogen and then stored at −80 °C until assayed for the number of bacteria per µL of DNA isolated from dental plaque (*P. gingivalis*, *Tannerella forsythia* (*T. forsythia*) and *Prevotella intermedia* (*P. intermedia*) (CFU)). Laboratory procedures for serum analyses and subgingival biofilm sample analyses are described in detail in Appendix A.

### 2.6. Laboratory Procedures

In vitro stimulation of cytokine production by circulating monocytes and myeloid dendritic cells.

A total of 500 μL of each peripheral blood sample was diluted 1/1 (vol/vol), in duplicate, in RPMI-1640 medium (Gibco; Paisley, Scotland, UK), supplemented with 2 mM L-glutamine and incubated at 37 °C, in a sterile environment, with a 5% CO_2_ humid atmosphere for 6 h, in the presence of 10 μg/mL of Brefeldin A (Sigma, St. Louis, MO, USA) to prevent the release of cytokines outside the cells. In addition, 100 ng/mL of lipopolysaccharide (LPS) from *Escherichia coli* (serotype 055:B5 (Sigma)) plus 100 U/mL of interferon-γ (IFN-γ) (Promega, Madison, WI, USA) were added to one of the tubes (stimulated samples). The other tube was used as a control to assess the basal production of cytokines (unstimulated sample).

### 2.7. Immunofluorescent Staining

After this incubation period, both stimulated and unstimulated samples were aliquoted in different tubes (200 μL/tube) to analyze the expression of each cytokine under study (TNF-α and IL-6) by the monocytes and myeloid dendritic cells (mDCs).

Immunophenotypic analysis was performed by using a six-color monoclonal antibody (mAbs) combination, detailed in Supplementary Table S1 (tube 1). We followed the protocol previously described in Martiín-Sierra et al., 2019 [26]. Briefly, the samples were aliquoted (200 μL) and stained with the mAbs for surface protein antigens (CD45, HLA-DR, CD16, CD33 and CD14). After extracellular staining, we proceeded with the intracellular staining for IL-6 or TNF-α cytokines, using the Fix & Perm kit (Life Technologies, Carlsbad, CA, USA), and following the manufacturer’s instructions.

### 2.8. Flow Cytometry Data Acquisition and Analysis

The stained samples were acquired in a FACSCanto II flow cytometer (BD, San Jose, CA, USA) and the data were analyzed with Infinicyt 2.0.6 software (Cytognos SL, Salamanca, Spain).

**Figure 1:**
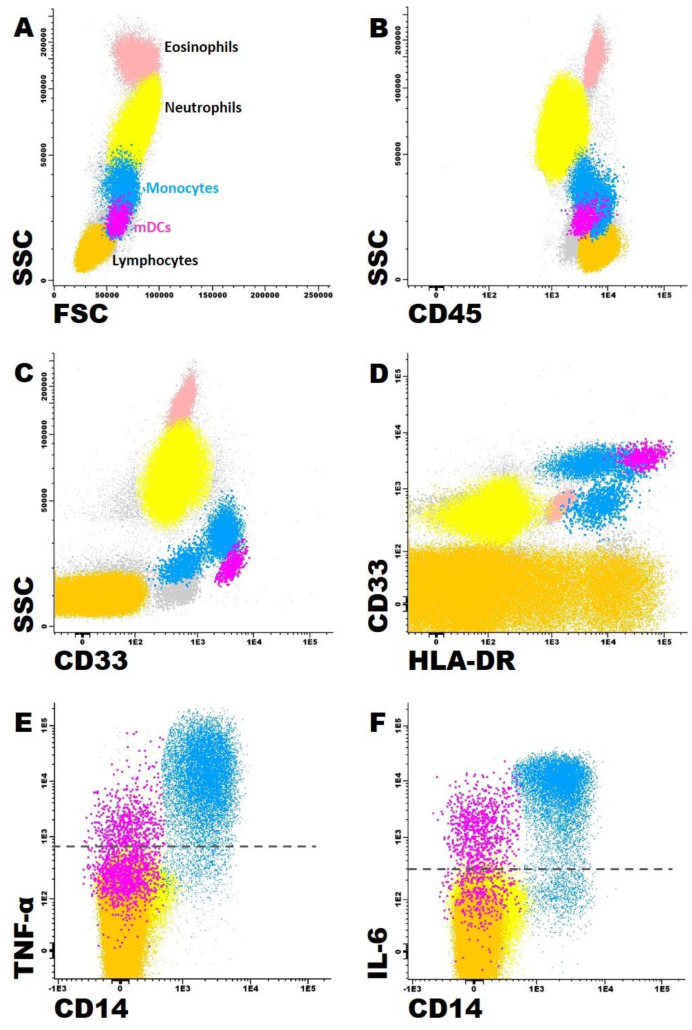
Gating strategy to identify peripheral blood monocytes and myeloid dendritic cells (mDCs). Monocytes (blue events) were identified based on their typical FSC/SSC characteristics (**A**), which lay between neutrophils (yellow events) and lymphocytes (identified in orange), together with high expression of CD45 (**B**), CD33 (**C**), HLA-DR (**D**) and CD14 (**E**,**F**). The mDCs (pink events) presented FSC/SSC values between those of monocytes and lymphocytes (**A**), along with lower levels of CD45 (**B**), and higher levels of CD33 (**C**) and HLA-DR (**D**), compared to monocytes; mDCs do not express CD14 (**E**,**F**). The percentages of monocytes and mDCs producing TNF-α (**E**) or IL-6 (**F**) were also evaluated. Light pink events correspond to eosinophils and gray events correspond to the remaining (non-identified) peripheral blood cells.

For the identification of monocytes, we used the following gating strategy: we selected the monocyte population by its characteristic FSC/SSC light dispersion properties, together with high expression of CD45, HLA-DR and CD33 (as displayed in Figure 1). The mDCs presented FSC/SSC values between those of monocytes and lymphocytes. They were further identified by their negativity for CD14 and CD16, showing higher levels of CD33 and HLA-DR, together with lower levels of CD45, as compared to monocytes.

After monocyte and mDC identification, the percentages of IL-6- or TNF-α-producing cells were evaluated, along with the mean fluorescence intensity (MFI) for each cytokine, which is a measure of the amount of protein produced per cell (Figure 1).

### 2.9. Statistics

Descriptive statistics were performed to describe the assessed variables. The assumptions required for parametric tests were evaluated using the Shapiro–Wilk test and Levene’s test. The Shapiro–Wilk test was employed to assess the normality of the data distributions, while Levene’s test was used to evaluate the homogeneity of variances across groups. The results indicated that the assumptions of normality and homogeneity of variance were not met. Consequently, non-parametric statistical tests were applied for further analysis. Continuous data comparisons were conducted using Mann–Whitney U test when 2 groups were involved, and Kruskal–Wallis for k samples was used for multiple comparison, followed by Dunn’s post hoc test procedure with a Bonferroni correction for multiple comparisons. Spearman’s correlation was used to test for statistical dependence between two variables. Data analysis was performed in SPSS v26.0 and R Studio v2023.09.1+494.

## 3. Results

### 3.1. Patients’ Characteristics

A total of 37 individuals were included in the study. The demographic data, clinical rheumatologic and periodontal parameters and laboratory findings across the three groups are presented in Table 1. All the patients were matched for age and gender.

The CRP levels showed statistically significant differences among the groups (*p* = 0.010, Kruskal–Wallis test). The HEALTHY group had a median CRP level of 1.57 mg/L, while the PERIO group had a median of 1.78 mg/L. Unsurprisingly, the PERIO+RA group had higher median CRP levels, of 3.63 mg/L.

No significant differences in PAD2_Act_ were found across the groups. When analyzing the PAD4_Act_, significant differences were observed between the HEALTHY (median of 28804.56 AU) and PERIO+RA (median of 40471.99 AU) groups (*p* = 0.001).

The anti-CCP2 IgG AU levels varied significantly (*p* < 0.001) among the groups. The HEALTHY and PERIO groups had medians of 1.65 AU and 1.42 AU, respectively, with all the individuals being negative for anti-CCP2 IgG, while the PERIO+RA group had a considerably higher median, of 44.65 AU. In the post hoc test, the PERIO+RA appeared to be significantly different from the HEALTHY and PERIO group: PERIO+RA vs. HEALTHY (*p* < 0.001); PERIO+RA vs. PERIO (*p* = 0.003).

No differences were observed between the PERIO and PERIO+RA groups regarding the periodontal parameters.

Statistically significant differences were found in the abundance of *T. forsythia* between the PERIO and PERIO+RA groups (*p* < 0.001).

Regarding the immune response to gingipains, although the PERIO group presented higher levels of anti-RgpB levels, no statistically significant differences were found between the groups. The anti-Kgp IgG levels were significantly higher in the PERIO+RA group compared to the HEALTHY controls (*p* = 0.050) and in the PERIO group compared with the HEALTHY group (*p* = 0.025).

**Table 1:** Clinical and laboratorial characteristics of the individuals included in the study.

	HEALTHY (*n* = 10)	PERIO (*n* = 10)	PERIO+RA (*n* = 17)	Difference Test
Age (years); median (IQR)	57.50 (5.00)	61.00 (8.00)	60.00 (31.00)	*p* = 0.351 ^(a)^
Male, % (N)	40.00% (4)	40.00% (4)	35.29% (6)	NA
CRP (mg/L); median (IQR)	1.57 (1.94)	1.78 (1.29)	3.63 (8.31)	*p* = 0.010 ^# (a)^
PAD2 activity (AU); median (IQR)	9254.41 (3003.08)	10,288.53 (2990.20)	9607.18 (1993.03)	*p* = 0.521 ^(a)^
PAD4 activity (AU); median (IQR)	28,804.56 (5650.33)	32,874.34 (10,135.41)	40,471.99 (22,299.97)	*p* = 0.001 ^## (a)^
DAS28-CRP; median (IQR)	NA	NA	2.36 (2.14)	NA
Anti-CCP2 IgG AU; median (IQR)	1.65 (1.70)	1.42 (4.04)	44.65 (540.82)	*p* <0.001 ^### (a)^
RA therapy				
csDMARDs; % (N)	NA	NA	94.12%; (16) 6 Methotrexate only 1 Leflunomide only 1 Salazopyrin only 3 Methotrexate + Hydroxychloroquine 1 Methotrexate + Leflunomide 1 Methotrexate + Sulfasalazine 2 Methotrexate + Hydroxychloroquine + Sulfasalazine 1 Sulfasalazine + Leflunomide	NA
Periodontitis				
Stage II grade B (N)	NA	0	2 Gen	NA
Stage III grade B (N)	NA	1 Loc; 5 Gen	4 Loc; 2 Gen	NA
Stage IV grade B (N)	NA	2 Gen	6 Gen	NA
Stage IV grade C (N)	NA	2 Gen	3 Gen	NA
Mean PD (mm); median (IQR)	NA	3.05 (1.33)	3.10 (0.85)	*p* = 1.000 ^(b)^
Mean CAL (mm); median (IQR)	NA	3.30 (3.15)	3.80 (1.85)	*p* = 0.421 ^(b)^
Maximum PD (mm); median (IQR)	NA	6.00 (2.00)	6.00 (2.00)	*p* = 0.059 ^(b)^
Maximum CAL (mm); median (IQR)	NA	6.50 (2.00)	7.00 (1.00)	*p* = 0.695 ^(b)^
BoP (%); median (IQR)	NA	44.50 (35.00)	44.00 (31.00)	*p* = 0.960 ^(b)^
PI (%); median (IQR)	NA	95.50 (28.00)	100.00 (13.00)	*p* = 0.052 ^(b)^
PISA (mm^2^); median (IQR)	NA	603.60 (491.75)	508.10 (934.95)	*p* = 0.547 ^(b)^
*P. gingivalis* CFU (AU); median (IQR)	NA	69,510,040.00 (113,308,278.00)	41,538,657.10 (85,053,344.84)	*p* = 0.177 ^(b)^
*T. forsythia* CFU (AU); median (IQR)	NA	144,441,559.50 (132,924,713.80)	27,981,448.19 (28,824,916.76)	*p* < 0.001 ^(b)^
*P. intermedia* CFU (AU); median (IQR)	NA	23,316,940.08 (35,611,641.55)	32,127,107.24 (66,825,460.65)	*p* = 0.902 ^(b)^
Anti-RgpB IgG; median (IQR)	267.30 (252.20)	507.10 (761.35)	261.36 (471.05)	*p* = 0.099 ^(a)^
Anti-Kgp IgG; median (IQR)	107.66 (220.04)	823.93 (976.30)	423.73 (1015.47)	*p* = 0.017 ^#### (a)^

AU = arbitrary units; BoP = bleeding on probing; CAL = clinical attachment loss; CCP2 = second-generation cyclic citrullinated peptide; CFU = colony-forming units; CRP = C-reactive protein; csDMARDs = conventional synthetic disease-modifying antirheumatic drugs; DAS28 = disease activity score for 28 joints; Gen = generalized; IQR = interquartile range; Kgp = lysine-specific gingipain; Loc. = localized; N = number; NA = non-applicable; PAD = peptidylarginine deiminase; *P. intermedia* = *Prevotella intermedia*; PD = probing depth; PI = plaque index; PISA = periodontal inflamed surface area; *P. gingivalis* = *Porphyromonas gingivalis*; RA = rheumatoid arthritis; RgpB = arginine-specific gingipain; *T. forsythia* = *Tannerella forsythia*; ^(a)^ = Kruskal–Wallis for k samples, assuming normality not verified; ^(b)^ = Mann–Whitney U test, assuming independent samples and normality not verified; ^#^ = differences between HEALTHY and PERIO+RA (*p* = 0.032); Differences between PERIO and PERIO+RA (*p* = 0.044); ^##^ = differences between HEALTHY and PERIO+RA (*p* < 0.001); ^###^ = differences between HEALTHY and PERIO+RA (*p* < 0.001) and between PERIO and PERIO+RA (*p* = 0.003); ^####^ = differences between HEALTHY and PERIO groups (*p* = 0.025) and between HEALTHY and PERIO+RA groups (*p* = 0.050).

### 3.2. Relative Quantification of Peripheral Blood Monocytes and Myeloid Dendritic Cells

From the data obtained by flow cytometry immunophenotyping, there were no differences regarding the frequency of peripheral blood monocytes between the groups. Concerning the frequency of mDCs in the whole blood, there were statistically significant differences between the HEALTHY and PERIO+RA groups, and the values were higher in the control group (*p* = 0.028).

### 3.3. Functional Alterations on Peripheral Blood Monocytes and Myeloid Dendritic Cells

#### 3.3.1. Frequency of Monocytes Producing IL-6 and TNF-α

The frequency of monocytes producing IL-6 or TNF-α, before and after activation with LPS and IFN-γ, did not show statistically significant differences between the studied groups (Table 2).

Exploring the PERIO+RA group, we found that five out of the seventeen included patients displayed statistically significant lower frequencies of IL-6-producing monocytes (and not TNF-α) after activation (*p* < 0.001). The comparative analysis of those patients revealed that the group of patients with the lowest frequency of monocytes producing IL-6 presented statistically significantly higher PAD 2 and PAD 4 activity, higher anti-RgpB IgG levels, higher mean PD, higher PISA and higher BoP. No differences were found for the other parameters.

#### 3.3.2. Frequency of Myeloid Dendritic Cells Producing IL-6 or TNF-α

No differences in the frequency of mDCs producing IL-6 or TNF-α were observed before LPS and IFN-γ activation.

The frequency of IL-6-producing myeloid dendritic cells after activation with LPS and IFN-γ did not show statistically significant differences between the studied groups (Table 2).

The frequency of TNF-α-producing myeloid dendritic cells, after activation with LPS and IFN-γ, presented statistically significant differences between the HEALTHY (median of 21.80%) and the PERIO+RA (median of 48.90) (*p* = 0.003). The PERIO group presented a median of 44.80%, but did not reach statistical significance (Table 2).

#### 3.3.3. Expression of IL-6 and TNF-α by Monocytes

Before stimulation with LPS and IFN-γ, the PERIO group showed higher monocyte protein expression of IL-6 (measured as MFI) (median of 2088.00) compared to the other two groups, presenting statistically significant differences when compared to the HEALTHY group (median of 1214.50) (*p* = 0.030) (Table 2).

After the LPS-and-IFN-γ stimulus, the PERIO group also presented a higher expression of IL-6 (MFI) by monocytes (median of 4634.00) compared to the other two groups, which was statistically significant between the PERIO and the PERIO+RA (median of 1316.00) (*p* = 0.008) (Table 2).

Before the stimulation with LPS and IFN-γ, there were no statistically significant differences between the studied groups for monocyte expression of TNF-α (Table 2).

After the LPS-and-IFN-γ stimulus, the monocytes from the PERIO group presented a statistically significantly higher expression of TNF-α (MFI) (median of 24,053.00) in comparison with the HEALTHY group (median of 5517.00) and the PERIO+RA group (median of 13,421.00) (*p* = 0.030 and *p* = 0.010, respectively) (Table 2).

#### 3.3.4. Myeloid Dendritic Cells’ Expression of IL-6 and TNF-α

No mDC expressions of IL-6 and TNF-α were observed before LPS and IFN-γ activation.

After stimulation with LPS and IFN-γ, although the mDCs from the PERIO group presented higher expressions of IL-6 (measured as MFI) compared to the other groups, there were no statistically significant differences among the groups (Table 2).

Regarding TNF-α expression by mDCs, the PERIO and PERIO+RA groups displayed higher values (medians of 8041.00 and 8230.00, respectively) compared to the HEALTHY group. There were statistically significant differences between the HEALTHY and PERIO groups (*p* = 0.014) and between the HEALTHY and the PERIO+RA (*p* = 0.003) (Table 2).

**Table 2:** Frequencies (%) of peripheral blood monocytes and myeloid dendric cells in the three studied groups. Frequencies (%) of TNF-α- and IL-6-producing peripheral blood monocytes and myeloid dendric cells and amount of each cytokine per cell (measured as MFI) after stimulation with LPS plus IFN-γ in the three studied groups.

			HEALTHY (*n* = 10)		PERIO (*n* = 10)		PERIO+RA (*n* = 17)		Differences between Groups ^(a)^	
			% of Cells	MFI	% of Cells	MFI	% of Cells	MFI	% of Cells	MFI
	% Monocytes (in whole blood); Median (IQR)		3.25 (1.25)	NA	3.53 (0.82)	NA	3.48 (2.78)	NA	*p* = 0.874	NA
Basal	IL-6	Total; Median (IQR)	4.82 (5.58)	1214.50 (394.25)	9.75 (14.71)	2088.00 (1134.50)	5.28 (12.25)	1611.00 (1069.50)	*p* = 0.285	*p* = 0.026 ^#^
	TNF-α	Total; Median (IQR)	2.47 (8.80)	3246.50 (1621.25)	8.92 (15.87)	7407.00 (9474.50)	5.07 (11.44)	3231.50 (1940.50)	*p* = 0.313	*p* = 0.350
Activated	IL-6	Total; Median (IQR)	74.05 (49.55)	1403.00 (602.00)	59.00 (24.80)	4634.00 (5178.75)	48.50 (83.63)	1316.00 (1569.05)	*p* = 0.554	*p* = 0.008 ^##^
	TNF-α	Total; Median (IQR)	67.80 (41.65)	5517.00 (5469.50)	85.30 (14.35)	24,053.00 (11,056.00)	82.60 (22.90)	13,421.00 (18,218.00)	*p* = 0.204	*p* = 0.007 ^###^
	% mDCs (in whole blood); Median (IQR)		0.13 (0.10)	NA	0.03 (0.11)	NA	0.04 (0.04)	NA	*p* = 0.032 ^####^	NA
Activated	IL-6	Total; Median (IQR)	40.90 (42.00)	1040.00 (433.75)	32.85 (47.45)	1894.50 (1476.75)	12.80 (52.50)	672.00 (652.00)	*p* = 0.373	*p* = 0.109
	TNF-α	Total; Median (IQR)	21.80 (29.50)	3645.00 (1998.00)	44.80 (31.35)	8041.00 (5192.00)	48.90 (22.45)	8230.00 (7970.50)	*p* = 0.004 ^#####^	*p* = 0.002 ^######^

IL-6 = interleukin-6; IQR = interquartile range; mDCs = myeloid dendritic cells; MFI = mean fluorescence intensity of positive cells; NA = non-applicable; TNF-α = tumor necrosis factor-α; % = percentage of positive cells; ^(a)^ = Kruskal–Wallis for k samples; ^#^ = differences between HEALTHY and PERIO (*p* = 0.030); ^##^ = differences between PERIO and PERIO+RA (*p* = 0.008); ^###^ = differences between HEALTHY and PERIO (*p* = 0.030) and between HEALTHY and PERIO+RA (*p* = 0.010); ^####^ = differences between HEALTHY and PERIO+RA (*p* = 0.028); ^#####^ = differences between HEALTHY and PERIO+RA (*p* = 0.003); ^######^ = differences between HEALTHY and PERIO (*p* = 0.014) and between HEALTHY and PERIO+RA (*p* = 0.003).

### 3.4. The Impact of the Studied Clinical, Microbiological and Serological Parameters on Frequency and Function of Monocytes and Dendritic Cells

**Figure 2:**
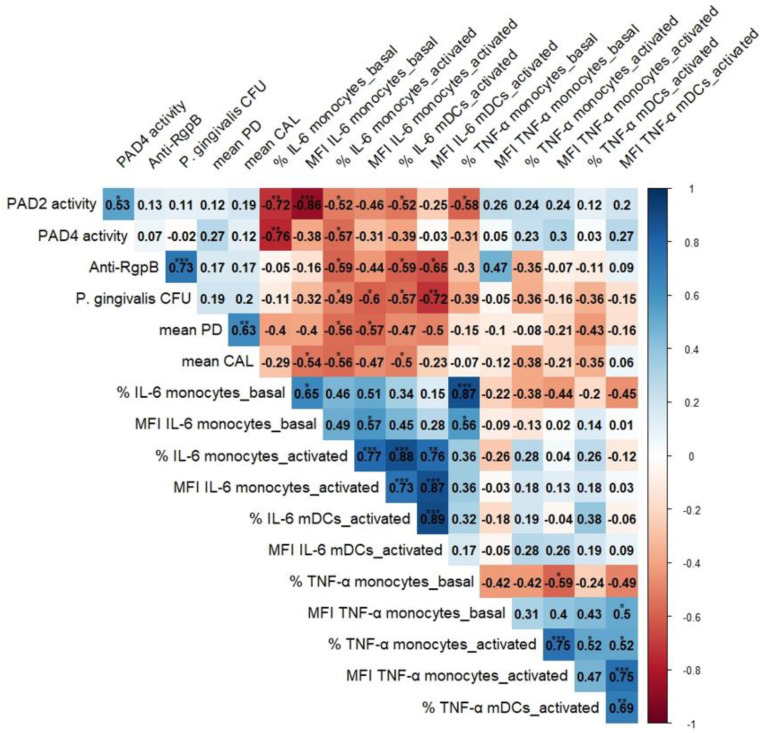
Graphical representation of the correlation matrix of the frequency and function of IL-6 and monocyte and dendritic cell producers of TNF-α and PAD2/4 activity, anti-RgpB IgG, *P. gingivalis* CFU, mean PD and mean CAL. The figure shows correlation coefficients r (scale on the right); p-values are shown as asterisks (* < 0.05; ** < 0.01; *** < 0.001). CAL = clinical attachment loss; CFU = colony-forming units; IL-6 = interleukin-6; mDCs = myeloid dendritic cells; MFI = mean fluorescence intensity of positive cells; PAD = peptidylarginine-deiminase; PD = probing depth; *P. gingivalis* = *Porphyromonas gingivalis*; RgpB = arginine-specific gingipain; TNF-α = tumor necrosis factor-α; % = percentage of positive cells.

To investigate whether there were any specific clinical, microbiological or serological characteristics that could have explained the obtained results, we further performed Spearman correlation analyses on the PERIO+RA (Figure 2) and PERIO groups.

In the PERIO+RA group, the analyses revealed that PAD 2_Act_ was negatively correlated with the frequency of IL-6-producing monocytes (before and after stimulus) (r = −0.723; *p* = 0.003; r = −0.520; *p* = 0.033, respectively). In the PERIO group, PAD 2_Act_ was also negatively correlated with the frequency of IL-6-producing monocytes before stimulus (r = −0.817; *p* = 0.007).

The PAD2 activity in the PERIO+RA group was also negatively correlated with the frequency of IL-6-produing mDCs after stimulus (r = −0.522; *p* = 0.031), and this correlation was also observed in the PERIO group (r = −0.636; *p* = 0.048). Regarding TNF-α, PAD 2_Act_ was negatively correlated with the frequency of TNF-α-producing monocytes before stimulus in the PERIO+RA group (r = −0.582; *p* = 0.018) and in the PERIO group (r = −0.733, *p* = 0.025).

The PAD4 activity was negatively correlated with the frequency of IL-6-producing monocytes (before and after stimulus) in the PERIO+RA group (r = −0.758; *p* = 0.002; r = −0.569; *p* = 0.017) and also in PERIO group, but only before stimulus (r = −0.833, *p* = 0.005).

In the PERIO+RA group, the anti-RgpB IgG levels were negatively correlated with the frequency of IL-6-producing monocytes after stimulus (r = −0.588, *p* = 0.013). In this group, the anti-RgpB levels were also negatively correlated with the frequency of IL-6-producing mDCs (r = −0.594, *p* = 0.012) and the mDC expression of IL-6 (r = −0.650, *p* = 0.009).

*P. gingivalis* CFU was negatively correlated with the frequency of IL-6-producing monocytes (after stimulus) (r = −0.494, *p* = 0.044) and with the monocyte expression of IL-6 (r = −0.600, *p* = 0.011) in the PERIO+RA group. Within this group, *P. gingivalis* CFU was also negatively correlated with the frequency of IL-6-producing mDCs (r = −0.565, *p* = 0.018) and the mDC expression of IL-6 (r = −0.720, *p* = 0.002).

In the PERIO+RA group, the mean PD was negatively correlated with the frequency of IL-6-producing monocytes after stimulus (r = −0.556, *p* = 0.020), and also with the monocyte expression of IL-6 after stimulus (r = −0.575, *p* = 0.016).

In the PERIO+RA group, the mean CAL was negatively correlated with the frequency of IL-6-producing monocytes after stimulus (r = −0.560, *p* = 0.019) and with the monocyte expression of IL-6 before stimulus (r = −0.536; *p* = 0.048). Regarding the myeloid dendritic cells, the mean CAL was negatively correlated with the frequency of IL-6-producing mDCs after stimulus (r = −0.503, *p* = 0.040).

### 3.5. The Impact of Periodontitis Severity on Frequency and Function of Monocytes and Dendritic Cells in Patients with Periodontitis and RA

In order to investigate whether the severity of periodontitis had an effect regarding the frequency of cells and the quantity of produced cytokines within the PERIO+RA group, the patients were split into two groups, according to periodontitis stage and extent, using the rationale developed by Eickholz et al. [27]: moderate periodontitis (stage II and localized stage III, *n* = 6) and severe periodontitis (generalized stage III and stage IV, *n* = 11).

We found that the severe periodontitis group presented a statistically significantly lower frequency of IL-6-producing monocytes and IL-6 expression by these cells (after stimulus) (*p* = 0.001 and *p* = 0.10, respectively) compared to the moderate periodontitis group. Regarding mDCs, we observed that the frequency of IL-6-producing mDCs and IL-6 expression by mDCs (after stimulus) was lower when compared to the moderate periodontitis group (*p* = 0.003 and *p* = 0.036, respectively).

## 4. Discussion

The aim of the present study was to quantify and characterize, by multi-color flow cytometry, peripheral blood monocytes and myeloid dendritic cells between groups (healthy, periodontitis and periodontitis plus RA). We further investigated the frequencies of TNF-α- and IL-6-producing peripheral blood monocytes and myeloid dendritic cells and the amount of each cytokine produced per cell, before and after stimulation with LPS plus IFN-γ. Further, a Spearman correlation analysis was performed in order to understand whether these quantifications were related to any clinical, microbiological or serological characteristics.

We found no differences regarding the frequency of peripheral blood monocytes between the groups. We observed a higher frequency of mDCs in the whole blood in the HEALTHY group compared to the other groups, with a statistically significant difference noted between the HEALTHY and PERIO+RA groups (*p* = 0.028). Based on previous studies [28,29], we hypothesized that the lower frequency of mDCs in circulation observed in the individuals with periodontitis and periodontitis plus RA could be due to the specific migration of mDCs to sites of infection. Evidence exists that DCs actively mobilize in and out of the oral mucosal tissues at different stages of periodontal health and disease [30,31], particularly increasing in the lamina propria of periodontitis tissues. DCs infiltrate the peripheral tissues in the immature state, where they capture foreign bodies and pathogens, but also debris and apoptotic cells. Upon pathogen recognition and capture, immature DCs undergo maturation. Due to their ability to present antigens via both major histocompatibility complex (MHC) classes I and II, mature DCs are described as professional antigen-presenting cells (APCs). Mature DCs also acquire a high migratory profile [32], allowing their infiltration to lymphoid organs and peripheral tissues [33].

Regarding the frequency of monocytes and mDCs producing IL-6 or TNF-α, as well as the expression of IL-6 and TNF-α, we observed a trend of higher values for the PERIO group, lower values for the PERIO+RA group and, as expected, even lower values for the HEALTHY group. Indeed, patients with periodontitis display increased levels of IL-6 and TNF-α in serum [34], saliva [34] and gingival crevicular fluid [35]. IL-6 plays a key role in the pathogenesis of periodontitis by inducing osteoclast differentiation and, consequently, bone resorption and inhibiting bone formation [36]. IL-6 can prompt the formation of osteoclasts from precursors at low concentrations, but when present at a high concentration, it primarily stimulates the activation of mature osteoclasts [37]. In addition to its effect on osteoclasts, studies have demonstrated that IL-6 has a connection to the release and activation of metalloproteinases, which may cause pathological extracellular matrix breakdown in periodontitis patients whose IL-6 serum levels are higher than normal [38,39]. Moreover, IL-6 is an important cytokine involved in the regulation of the host response to bacterial infection [40,41]. TNF-α also contributes to the upregulation of osteoclastogenesis and the downregulation of osteoblastogenesis [42]. TNF-α can also induce the expression of other mediators that amplify or sustain the inflammatory response (e.g., prostaglandins), stimulate the production of lytic enzymes (e.g., collagenase), and enhance bacterial killing and phagocytic activity [43].

The lower frequencies of monocytes and mDCs producing IL-6 or TNF-α and the decreased expression of IL-6 and TNF-α in the PERIO+RA group may be partially explained by the fact that those patients were under RA therapy. Although we excluded patients under biological therapy in this study, we do know that conventional drugs for RA treatment, through various mechanisms of action, also suppress immune system factors or cytokines secreted by immune cells [44].

Exploring the PERIO+RA group, we found that a subgroup of the included patients displayed statistically significantly lower frequencies of monocytes producing IL-6, but not TNF-α, after activation. This subgroup of patients presented statistically significantly higher PAD 2 and PAD 4 activity, higher anti-RgpB IgG levels, higher mean PD, higher PISA and higher BoP. It was expected that the patients with periodontitis plus RA would demonstrate higher frequencies of monocytes and mDCs and related cytokine production. However, we observed the opposite. We hypothesize that worse periodontal inflammation, together with RA, may induce an overload and exhaustion of cytokine producing cells. Indeed, when analyzing the severe periodontitis group, compared to the moderate periodontitis group, we observed a statistically significantly lower frequency of IL-6-producing monocytes and mDCs, as well as monocyte and mDC expression of IL-6 (after stimulus). This hypothetical exhaustion of the innate response may, in turn, result in more severe periodontitis in patients with RA, since the disease may progress without immunity opposition.

Importantly, we found that anti-RgpB and *P. gingivalis* CFU were negatively correlated with the intracellular expression of IL-6 by monocytes and mDCs. RgpB, which is known to be one of the most potent virulent factors described for *P. gingivalis* [45], is capable of chemokine paralysis, ensuring that low-grade infection can continue unhampered [46]. Moreover, one key pathological consequence of *P. gingivalis* infection of dendritic cells is that it induces a state of immune senescence, to escape immune elimination [47], which is evidenced by defective maturation and immunostimulatory functions. This may result in the prolonged survival of *P. gingivalis*, possibly driving autoantibody formation and a self-perpetuating loop of chronic inflammation. Accordingly, we observed a negative correlation between PAD 2/4 activity and the frequency of monocyte and mDC producers of IL-6 and TNF-α, showing that PAD enzymes continue to promote citrullination and, consequently, ACPA formation, fueling RA.

Our cohort was very well-characterized according to the latest periodontal and rheumatological recommendations. The fact that we did not include patients under biological therapy and that we assured the stability of the RA medication 3 months before the inclusion reinforces the results’ reliability. Our study is, however, subject to several limitations, most notably the relatively small sample size and the cross-sectional design. These factors may constrain the generalizability of our findings. To confirm the associations that we have observed and to elucidate the causal relationships between *P. gingivalis* infection, periodontitis severity and monocyte/mDC frequency and function in patients with periodontitis and RA, longitudinal studies with larger cohorts are required.

## 5. Conclusions

In conclusion, our study demonstrated that periodontitis induces significant changes in the homeostasis of peripheral blood monocytes and mDCs, either in number or in their ability to produce pro-inflammatory cytokines. Our data support an immunologic link between *P. gingivalis*, periodontitis severity and RA. In patients with periodontitis plus RA, immune cells produce fewer cytokines, and this is correlated with periodontitis-related clinical and microbiological parameters. This could result in the impaired clearance and prolonged presence of *P. gingivalis* in RA patients, which may induce a low level of systemic inflammation, contributing to the perpetuation and/or severity of periodontitis and RA.

## Data Availability

The data presented in this study are available on request from the corresponding author due to ethical reasons.

## Supplementary Materials

The following supporting information can be downloaded at: https://www.mdpi.com/article/10.3390/jcm13175297/s1. Table S1: Panel of monoclonal antibody reagents (with clones and commercial source) used for the immunophenotypic characterization of monocytes and myeloid dendritic cells.

## Appendix A

Serum analyses:

CRP:

Serum CRP concentration was measured using a commercially available ELISA kit (RnD) according to manufacturer’s instructions.

Anti-CCP2 IgG:

Anti-CCP2 IgG was measured using the commercial Immunoscan CCPlus^®^ (Svar Life Science, Malmö, Sweden) kit according to the manufacturer’s protocol. The anti-CCP2 IgG levels of all study participants were included in the anti-CCP2 IgG analysis, even if the cut-off for positivity (≥25 AU) was not reached.

PAD2/4 activity:

To evaluate PAD 2 or PAD 4 activity levels in serum samples, 20 µL of serum were mixed with 1 mU of the respective recombinant enzyme (kindly provided by Dr Ewa Bielecka, Dr Alicja Sochaj-Gregorczyk and Dr Stanisław Malicki), 1 mM substrate (Dansyl-Gly-Arg), 2 mM o-phenantroline and 10 mM DTT. Reaction buffer (100 mM Tris, pH 7.5 with 10 mM CaCl_2_) was added to the final volume of 30 µL. Samples were then incubated for 60 min (PAD4) or 90 min (PAD2) at 37 °C. The reaction was stopped by the addition of 40 µL 5% ice-cold trichloroacetic acid. Proteins were allowed to precipitate during 15 min incubation on ice, and then 35 µL of 1% trifluoroacetic acid (TFA) was added. Samples were centrifuged for 10 min at maximum centrifuge speed in 4 °C. Supernatants were transferred to vials. High-performance liquid chromatography (HPLC) was performed using Shimadzu system LabSolutions CS Software version 5.71 SP2 (Shimadzu Co., Kyoto, Japan). In total, 50 µL of the sample were injected onto AERIS Peptide XB-C18 4.6/150 mm column (Phenomenex). After column equilibration, products of the reaction were eluted in a rising gradient of 10–35% of phase B (80% acetonitrile, 0.08% TFA). Dansyl substrate and the citrullinated product were detected at 330 nm excitation and 530 nm emission. The product peak area was then normalized to the reference serum sample and used to quantify serum PAD activity.

Anti-RgpB and anti-Kgp:

High-binding Nunc MaxiSorp plates were coated overnight at 4 °C with the RgpB protein or the full-length Kgp protein (2.5 μg/mL) in 50 mM carbonate buffer, washed in PBS-Tween (0.05%) and blocked (2% BSA in PBS) ON at 4 °C. Serum, diluted 1:800 in RIA-buffer (1% BSA, 350 mM NaCl, 10 mM Tris-HCl (pH 7.6), 1% Triton X-100, 0.1% SDS), was incubated for 1.5 h at room temperature (RT). Plates were then washed and incubated for 1 h at RT with peroxidase-conjugated goat anti-human IgG (diluted 1:10.000 in RIA-buffer). Bound antibodies were detected using tetramethylbenzidine substrate (Sigma). The color reaction was stopped with 0.5 M H_2_SO_4_. Absorbance was measured at 450 nm. Antibody levels were presented as arbitrary units (AU), based on a standard curve made from a serially diluted highly positive serum pool.

Analyses of subgingival biofilm samples:

DNA isolation

Plaque samples were centrifuged for 15 min at 10,000 rpm, 4 °C, and RNAlater was discarded. DNA was isolated using a commercially available QIAamp DNA mini kit (Qiagen, Hilden, Germany) according to the product’s instructions.

Real-time PCR:

Equal volumes of salivary DNA template (1 µL) were pipetted onto a 96-well plate. Next, 7.5 µL of PowerUP SYBR Green Master Mix 2× (Thermo Fisher, Waltham, MA, USA), 0.5 µL of forward and reverse primers (Table A1) were added (final primer concentration during reaction: 0.33 µM), and water was added to the final volume of 15 µL. PCR reaction was performed in the following conditions for 16S reactions: 2 min 95 °C, 40 cycles of 15 s 95 °C and 1 min 60 °C, with final elongation 2 min at 60 °C. Reaction-product quality was analyzed via melt curve and compared with positive controls run in parallel during every reaction.

Colony-forming units (CFU) of *P. gingivalis*, *T. forsythia* and *P. intermedia* were calculated using threshold values for standard curves obtained using DNA preparations of a known amount of bacteria.

**Table A1:** Oligonucleotide sequences of primers used in the study.

	Forward (5′->3′)	Reverse (5′->3′)
*P. gingivalis* 16S	AGGCAGCTTGCCATACTGCG	ACTGTTAGCAACTACCGATGT
*T. forsythia* 16S	GCGTATGTAACCTGCCCGCA	TGCTTCAGTGTCAGTTATACCT
*P. intermedia* 16S	GACCAAAGATTCATCGGTGGAG	CACGCTACTTGGCTGGTTC
