# Supplementary material for: Impaired Periodontitis-Induced Cytokine Production by Peripheral Blood Monocytes and Myeloid Dendritic Cells in Patients with Rheumatoid Arthritis: A Case–Control Study
Source: J Clin Med. 2024 Sep 6;13(17):5297. doi: 10.3390/jcm13175297 (PMC11395796; doi:10.3390/jcm13175297)
Supplement: Supplementary file 1 [file jcm-13-05297-s001.zip › jcm-3180147-supplementary.pdf]

Supplementary Table S1: Panel of monoclonal antibody reagents (with clones and commercial sources) used for the immunophenotypic characterization of monocytes and myeloid dendritic cells.

**Tube**	**PB**	**PO**	**PE**	**PE-Cy7**	**APC**	**APC-H7**
**1**	HLA-DR	CD45	cyTNF-α	CD16	CD33	CD14
**Clone**	L243	2D1	Mab11	3G8	P67.6	MφP9
**Commercial source**	BD	BD	BD	BD	BD	
**2**	HLA-DR	CD45	cyIL-6	CD16	CD33	CD14
**Clone**	L243	2D1	MQ2-6A3	3G8	P67.6	MφP9
**Commercial source**	BD	BD	BD Pharmingen	BD	BD	BD

APC = allophycocyanin; **APC-H7** = allophycocyanin-hilite 7; **cy** = cytoplasmic staining; **IL-6** = interleukin-6; **PE** = phycoerythrin; **PE-Cy7** = phycoerythrin-cyanine 7; **TNF-α** = tumor necrosis factor-α. **Commercial sources: BD** (Becton Dickinson Biosciences (BD), San Jose, CA, USA), **BD Pharmingen** (San Diego, CA, USA).
